# Conservation Value and Permeability of Neotropical Oil Palm Landscapes for Orchid Bees

**DOI:** 10.1371/journal.pone.0078523

**Published:** 2013-10-17

**Authors:** George Livingston, Shalene Jha, Andres Vega, Lawrence Gilbert

**Affiliations:** 1 Department of Integrative Biology, University of Texas at Austin, Austin, Texas, United States of America; 2 AMBICOR, Tibas, Costa Ruca; University of Western Ontario, Canada

## Abstract

The proliferation of oil palm plantations has led to dramatic changes in tropical landscapes across the globe. However, relatively little is known about the effects of oil palm expansion on biodiversity, especially in key ecosystem-service providing organisms like pollinators. Rapid land use change is exacerbated by limited knowledge of the mechanisms causing biodiversity decline in the tropics, particularly those involving landscape features. We examined these mechanisms by undertaking a survey of orchid bees, a well-known group of Neotropical pollinators, across forest and oil palm plantations in Costa Rica. We used chemical baits to survey the community in four regions: continuous forest sites, oil palm sites immediately adjacent to forest, oil palm sites 2km from forest, and oil palm sites greater than 5km from forest. We found that although orchid bees are present in all environments, orchid bee communities diverged across the gradient, and community richness, abundance, and similarity to forest declined as distance from forest increased. In addition, mean phylogenetic distance of the orchid bee community declined and was more clustered in oil palm. Community traits also differed with individuals in oil palm having shorter average tongue length and larger average geographic range size than those in the forest. Our results indicate two key features about Neotropical landscapes that contain oil palm: 1) oil palm is selectively permeable to orchid bees and 2) orchid bee communities in oil palm have distinct phylogenetic and trait structure compared to communities in forest. These results suggest that conservation and management efforts in oil palm-cultivating regions should focus on landscape features.

## Introduction

 The greatest threat to terrestrial biodiversity is land-use change [1]. Among drivers of land-use change, the most important is the expansion and intensification of agricultural land [2]. In recent decades, the majority of global agricultural expansion has occurred in tropical regions [3]. In particular, the expansion of international commodity agriculture (e.g., coffee [4], soybeans and oil palm [5]) in the tropics presents a major global conservation and sustainability challenge [6]. Oil palm (*Elaeis* spp.) is among the most important of these commodity crops in the wet tropics, covering 14.5 million hectares globally [7] and rapidly expanding due to demand as a key biofuel feedstock [8,9]. Despite its importance, less than 1% of published studies on oil palm examine biodiversity impacts and a majority of these do not involve field-based data [9]. Documenting and explaining patterns of biodiversity loss are a key first step towards developing more sustainable agricultural production [5]. 

 Three research foci related to biodiversity in oil palm require urgent attention [7]: 1) the local conservation value of oil palm, 2) the permeability between oil palm and tropical forests, and 3) the potential ecosystem services provided by oil-palm inhabiting organisms. The first concern, local conservation value of oil palm, is relatively well studied and is thought to be generally low relative to agroforestry [10] or traditional tropical gardens [11]. This is because oil palm harbors no forest tree species, lianas, epiphytic orchids, or indigenous palms [12] and this indirectly and usually negatively affects animal diversity [13]. Among 13 studies comparing animal taxa between forest and oil palm, species richness in oil palm was an average of 15% of that in forest, with reduced abundance and similarly low compositional overlap [13]. Still this number is likely an overestimate due to detection biases and possible extinction debts [13]. Wide variation exists among taxa, with some groups, like bees in Southeast Asia, showing greater richness in oil palm [14]. 

The second major concern for biodiversity within oil palms, the permeability of oil palm to dispersal from adjacent forest habitats, is addressed by only a few studies, though there is evidence of some permeability. Specifically, landscape-level forest cover has been associated with slight increases in bird and butterfly richness in oil palm [15] and studies that have directly examined permeability of oil palm to butterflies and ants have observed a reduction in species richness and a decay in community similarity with distance from forest [16]. The third concern, the impact of biodiversity on ecosystem services in oil palm, is also understudied; however, there is evidence for a positive role for biodiversity in biocontrol, pollination, and decomposition in oil palm [7,15,17]. However, much more work is needed to fully address these three basic questions in oil palm [7], especially in the Neotropics, where oil palm has the potential for rapid future expansion [18]. 

 In this study, we examine the local conservation value and permeability of oil palm using orchid bees (Euglossini) in Southwestern Costa Rica. We sampled orchid bee communities in four regions; 1) a forest of high conservation value (Parque Nacional Corcovado), 2) an oil palm plantation immediately adjacent to forest, 3) an oil palm plantation greater than 2 km from forest and 4) an oil palm plantation greater than 5 km from forest. Orchid bees are among the most well-known tropical insects and have a well-resolved phylogeny with trait data available for many species [19-21]. The availability of the phylogeny makes it possible to calculate metrics of community phylogenetics [22], where such metrics can serve as indicators of underlying community assembly mechanisms. In situations where species’ traits are phylogenetically conserved, it is possible to identify cases where the community may have assembled via competitive sorting into diverse niches versus scenarios where environmental filtering into few niches played a stronger role [23]. 

Orchid bees are major pollinators of many families of tropical plants [19]. Like most bees, orchid bees are dependent on the availability of nectar and nest-site resources [24]. In addition, many species are thought to be dependent on a wide array of orchid and non-orchid derived perfume sources for mating behaviors [25]. It is believed that collecting perfume sources drives long-distance foraging in orchid bees [26], making the group especially interesting for studying permeability. 

 Given the complex natural history of orchid bees, their long-distance movement, and the absence of most forest plant species in oil palm, we hypothesized that orchid bees would be sensitive to landscape conversion to oil palm. Specifically, we considered two main hypotheses: 1) Species richness, abundance and community similarity of orchid bees in oil palm decline with increasing distance from forest and 2) changes in community composition are associated with altered community phylogenetic and trait structure. 

## Methods

### Study sites

**Figure 1:**
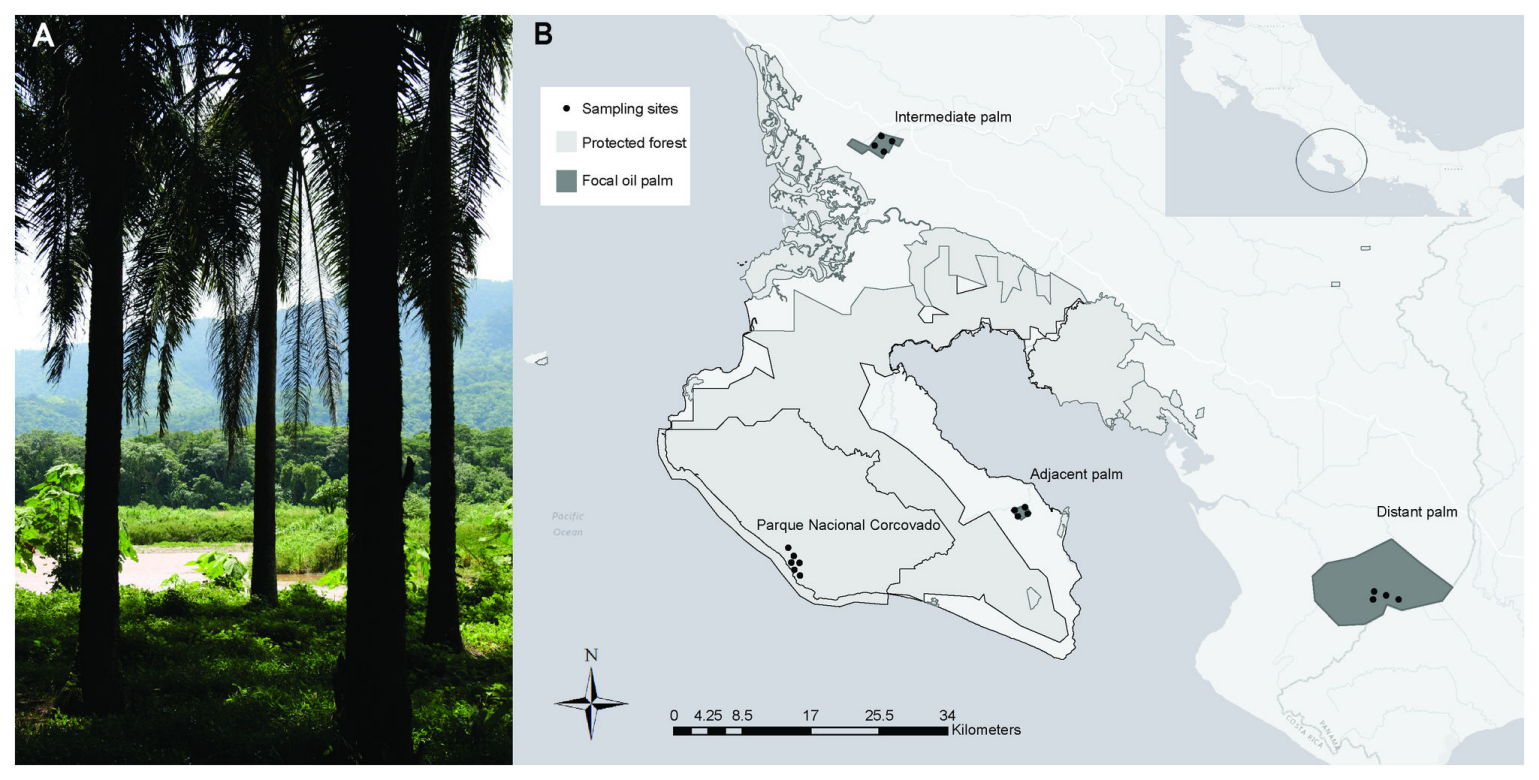
Image and Map of the study region. (A) An interface of oil palm and adjacent forested areas (Photo Credit: GL). (B) The Osa Peninsula including areas of protected forest and the focal oil palm regions.

 We surveyed the orchid bee community in four regions; 1) forest, 2) oil palm sites adjacent to the forest (0.5-1.365 km), 3) oil palm sites at intermediate distance from the forest (2.308-3.638 km), and 4) oil palm sites far from forest (5.394-6.930 km) (Figure 1). The dominant land cover in each region was either forest or oil palm such that as distance from forest increased the area of regional forest cover progressively decreased (calculated within a 5km radius using Google Earth, Table 1). The study took place during the onset of the wet season in June and July 2012. The forest included sites surrounding the Sirena Station in Parque Nacional Corcovado (44,485-hectares) on the Osa Peninsula (8° 28’ N and 83° 35’ W). This area was previously sampled in 1977 [27]. The adjacent oil palm sites were within a 10 year-old 150-hectare plantation operated by Palma Tica located adjacent to Puerto Jimenez (Osa Peninsula, 8° 32’ N and 83° 20’ W). The intermediate oil palm sites were within a 25 year-old 685-hectare plantation operated by Palma Tica (mainland near Palmar Norte, 8° 56’ N and 83° 29’ W). The distant oil palm sites were within a 10,000-hectare 20-25 year-old group of plantation blocks operated by COOPEAGROPAL (mainland near Laurel, 8° 26’ N and 82° 56’ W). Management practices employed by Palma Tica and COOPEAGROPAL are similar and involve the use of insecticides and herbicides. No insecticides were applied within one week of sampling and herbicides were applied continuously. 

**Table 1:** Environmental variables among regions.

Region	Percent forest cover	Relative humidity	Temp opening (C)	Temp closing (C)	Palm height (m)	Percent canopy cover	Percent epiphyte cover	Inflorescences
**Forest (n=7)**	100	93.67 (2.7)	28 (0.4)	29.2 (0.65)	-	-	-	-
**Adjacent palm (n=4)**	63	83.9 (4.0)	27.98 (0.4)	30.22 (0.95)	8.9 (0.64)	90 (0.01)	49 (1.3)	15 (4.3)
**Intermediate palm (n=4)**	31	80 (3.2)	27.68 (0.36)	30.73 (0.68)	9.3 (0.49)	86 (0.01)	93 (3.1)	13 (4.5)
**Distant palm (n=4)**	1	82.23 (4.0)	25.73 (0.18)	30.05 (0.66)	10.1 (0.14)	83 (0.02)	93 (2.6)	19 (6.1)

Values show means across sites and () show standard error. Percent forest cover is calculated only from the centroid of each region.

### Orchid bee sampling

 We sampled multiple sites within each region, and all sites were separated by 1km in all directions. We sampled the forest more intensively because of its much greater structural complexity and potential as the ultimate source habitat for bee species in oil palm. Seven sites were sampled in the forest region and four sites in each of the different plantation regions. Sites in the forest were placed adjoining light gaps based on ease of access to trails and similar elevation. Sites in oil palm were placed at the center of each region. Each region was sampled over a seven or four day period and each site within a region was sampled for one day. We did not sample sites repeatedly because our destructive sampling procedure could significantly impact local population sizes [28]. We recorded temperature and relative humidity at all sites, and at oil palm sites we recorded five additional environmental variables: 1) distance from nearest continuous forest edge using GPS coordinates and Google Earth, 2) palm height using a rangefinder reported as the average from 10 trunks, 3) canopy cover using non-hemispherical digital photos and ImageJ, reported as the average from three randomly selected locations, 4) percent epiphyte cover on 10 trunks using digital photos, and 5) inflorescence abundance within a 20 meter diameter circle centered on the site. Permission to collect orchid bees and permission to sample in Parque Nacional Corcovado was obtained from the Ministerio del Ambiente y Energía (Permit # 099-2012-SWAC). Permission to sample in the plantations was obtained by Palma Tica and COOPEAGROPAL. This study did not involve endangered or protected species. 

We used chemical baits to survey male orchid bees. This method is widely used to infer orchid bee population and community structure [26-29]. Baits were cotton balls soaked in mineral oil to which we added six drops of either methyl salicylate or cineole. These two attractants together capture the greatest diversity of orchid bee species in Southwestern Costa Rica [28]. The use of mineral oil reduces the rate of volatilization [30] helping to ensure that the odor plume intercepts foraging bees from a localized area. These baits were placed in four custom-made cone-style traps with a bow of blue, pink, and red flagging as a visual lure. The four traps were placed 10 m distant in a square pattern. We modified the timing of trap exposure slightly to minimize the potential for thermal differences to confound our results. Specifically, in the forest, traps were opened at 900 hr and closed at 1300 hr, a period corresponding to maximal orchid bee activity [31]. In the oil palm, where temperatures are higher [7], traps were opened at 800 hr and closed at 1300 hr to catch bees that may forage earlier in the day [31]. Captured bees were collected every 80 minutes and preserved in ethanol for counting and identification. After collection, we also determined three trait variables, either measured or surveyed from the literature [19]: 1) geographic range (the total number of biogeographic regions a species is recorded to occur (maximum seven)), 2) tongue length (mm), and 3) body mass (mg). To assess phylogenetic community structure, we used a molecular phylogeny for all Euglossini [19]. Using the phylogeny, we calculated mean phylogenetic distance (MPD) only for the *Euglossa* community at each site. We focused on *Euglossa* because it accounted for the majority of species and individuals and is less likely to be affected by strong sampling biases [32]. High MPD scores indicate greater evenness (suggesting niche diversification assembly mechanisms), whereas low scores indicate clustering (suggesting environmental filtering) [22,33]. We used the K statistic to assess the strength of phylogenetic conservatism of traits [34].

### Statistical analyses

Due to the differing sampling efforts and catch sizes among regions, species richness was rarified to the smallest catch size at any site in each region. To test hypothesis one, we examined the correlation between Poisson-compound gamma estimated richness [35], mean capture rate per hour, and Cao community similarity to the forest community [36] with distance from forest. We descriptively examined species’ responses to distance to forest using PCA (abundances Hellinger transformed). To test hypothesis two and examine community trait and phylogenetic structure, we correlated distance from forest with MPD scores and all three traits. Significance of correlations was tested using one-way ANOVA. Richness was estimated using the SPECIES package [35], compositional analyses were carried out in the Vegan package [37] and phylogenetic analyses in the Picante package [33] in R (R Development Core Team 2013). 

## Results

**Figure 2:**
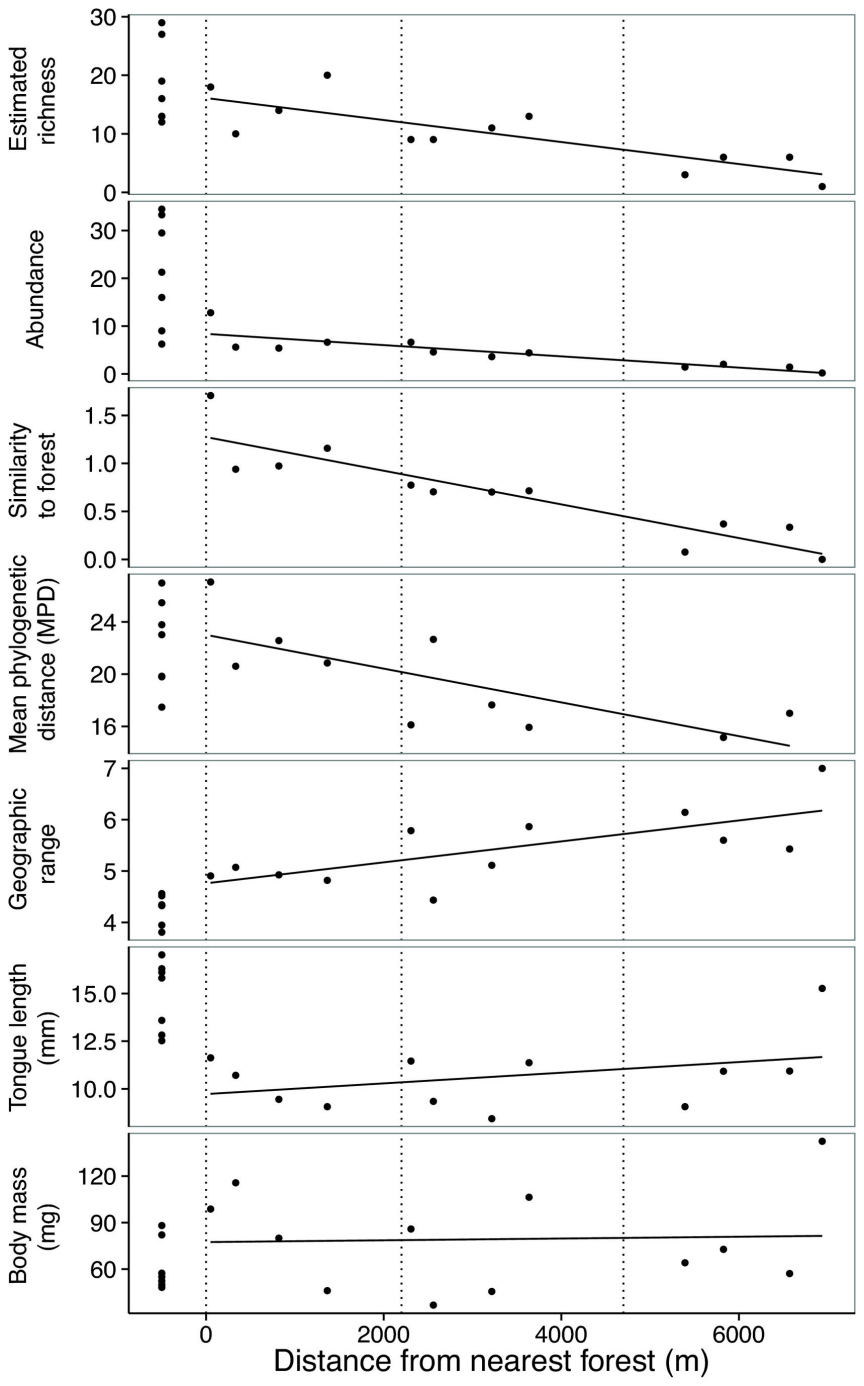
Distance from forest and community properties for all 19 sites. Regression lines are only fit to oil palm sites. Estimated richness is Poisson-compound gamma estimated (Y=-0.002x + 16.11, R^2^=0.66, F=18.87, df=10, P<0.01). Abundance is the mean hourly capture rate (Y=-0.001x + 8.38, Rsq=0.74, F=27.05, df=10, P<0.001). Similarity to forest is reported using the Cao method (Y=-0.0001x + 1.27, R^2^=0.81, F=43.08, df=10, P<0.0001). Mean phylogenetic distance is the observed scores for each site (Y=-0.001x + 23, R^2^=0.56, F=9.97, df=10, P<0.05). Geographic range represents the abundance-weighted mean number of biogeographic regions in which species occur (Y=0.0002x + 4.76, R^2^=0.50, F=10.06, df=10, P<0.01). Tongue length is the abundance-weighted mean across species (Y=0.0003x + 9.73, R^2^=0.14, F=1.63, df=10, P=0.23). Body mass is the abundance-weighted mean across species (Y=5.73E-4x + 77.44, R^2^=0.002, F=0.02, df=10, P=0.89).

Overall we caught 872 bees of 26 species (77% were caught on cineole, Table S1). Orchid bees were present in all regions but mean estimated species richness across forest sites was 18.43 (SE=2.64) and declined significantly with distance from forest to a low of 4 (SE=1.23) in the distant palm sites (P<0.01, Figure 2). Hourly capture rates showed a similar significant decline from 22.34 (SE=4.34) to 1.25 (SE=0.38) (P<0.001, Figure 2). The proportion of individuals captured during each collection period varied with time since opening but did not increase or decrease significantly (Figure S1). Generally, the plantation regions were similar in environmental conditions, oil palm was hotter and drier than forest, and no orchids were observed in oil palm (Table 1, [16]). 

**Figure 3:**
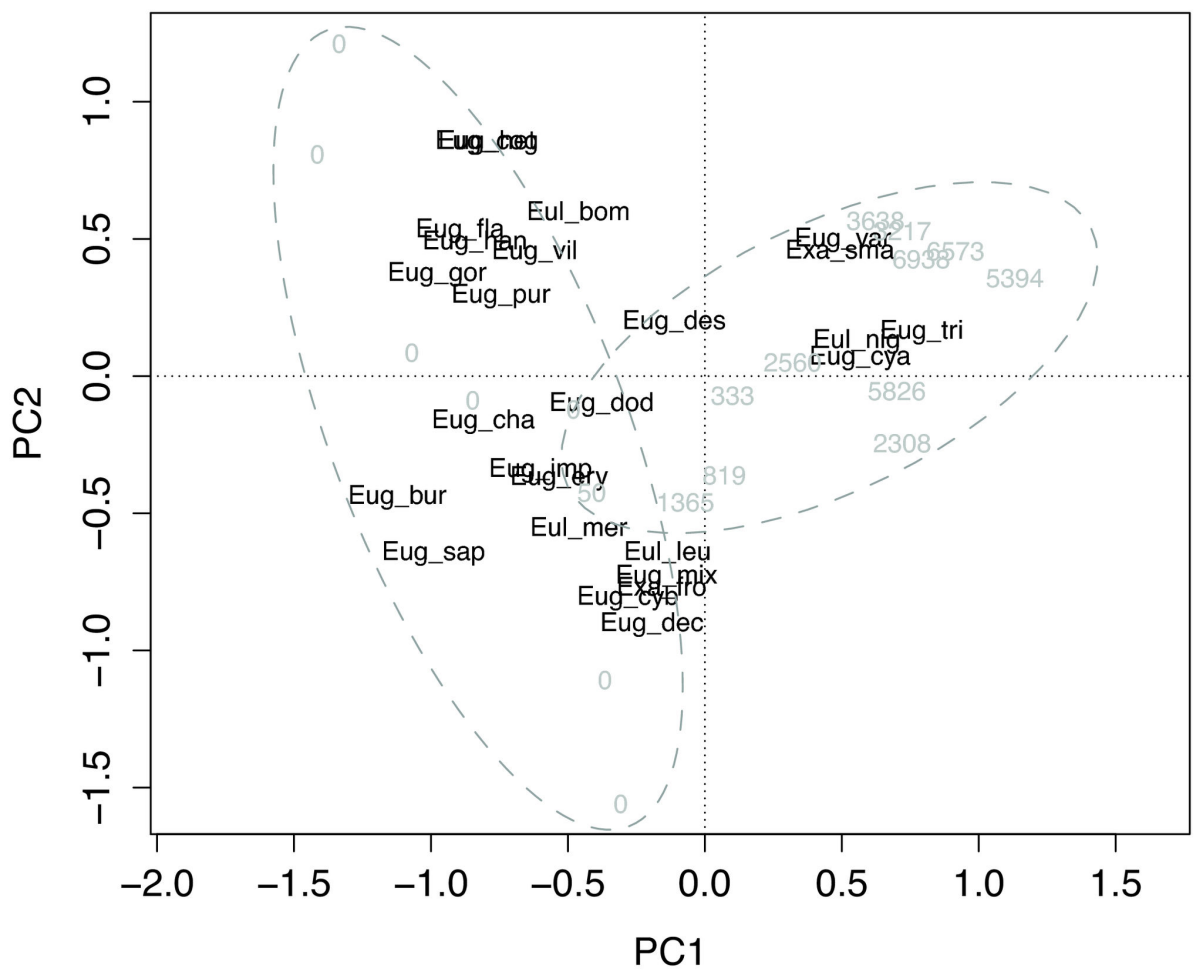
PCA plot showing the distinct responses of species to distance from forest. Sites are displayed as distance in meters from forest (0 for forest sites). Forest and oil palm sites are distinguished with dashed ellipses. Species names are abbreviated; full names can be found in Table S1 and S2. Species scores are standardized to unit variance.

 Species present in oil palm sites largely included those also found in the forest. Only two species were unique to oil palm (total individuals sampled=331), while seven were unique to the forest (total individuals sampled=548). Species relative abundances strongly diverged between forest and oil palm with the greatest divergence between forest sites and the distant oil palm sites (Figure 3). *Euglossa imperialis* was the dominant species in the forest, while in oil palm *Euglossa tridentata* became progressively more dominant with increasing distance from forest (Figure 3, Table S1 and S2). The second most dominant forest species, *Euglossa sapphirina*, also declined in oil palm, while the parasitic species, *Exaerete smaragdina*, became the second-most dominant in oil palm sites (Figure 3, Table S1 and S2). Similarity of the oil palm community and forest community declined significantly with increasing distance from forest (P<0.0001, Figure 2). 

 Mean phylogenetic distance (MPD) scores were higher in the forest and declined with increasing distance from forest (P<0.05, Figure 2). Among traits, average abundance-weighted community geographic range size was lower in forest and increased with distance from forest (P<0.01), tongue length was lower in oil palm (F=23.68, df=17, P<0.001) but was not significantly correlated with distance, and body mass showed no pattern (Figure 2). Using all species we captured, the K statistic indicated significant phylogenetic conservatism for tongue length (K=2.01, SD=0.61, N=20, P<0.01), but random patterns for range (K=0.66, SD=0.79, N=20, P=0.251) and body mass (K=0.87, SD=3.53, N=20, P=0.108). 

## Discussion

In this study we show that orchid bee communities are sensitive to oil palm habitat and increasing isolation from forest habitat. Our results support our first hypothesis that species richness, abundance and community similarity of orchid bees in oil palm decline with increasing distance from forest. This result is also consistent with observations for a wide range of taxa in Asian and African oil palm plantations [13]. However, unlike other studies that generally found low compositional overlap with forest (<50% [13]), we found that 90 percent of species in oil palm also occurred in Parque Nacional Corcovado. These results suggest that orchid bees move frequently between forest and oil palm or that some species are able to establish populations in both habitats. 

 The literature on the movement ecology of orchid bees is substantial, yet clear patterns remain unresolved [38]. Some studies have suggested male orchid bees do not regularly cross non-forested open areas [39], whereas others suggest they readily make such trips over tens of kilometers [40] and integrate multiple forest fragments into their foraging ranges [41]. Recently, the use of smaller quantities of attractant and micro radio-telemetry suggests that foraging home ranges are smaller than once thought, possibly 42-115 hectares [38]. Although we cannot estimate precise movement patterns or distances, we used small quantities of attractant and mineral oil to slow the release rate and our capture rate suggests our traps are intercepting locally foraging bees relative to some previous studies [28]; thus, our results likely reflect true gradients in the density and composition of foraging orchid bees. 

 Our distance-decay in similarity and declining abundance results suggest substantial permeability of oil palm to some orchid bee species [16,42]. The observation that there is a significant linear decline in similarity to forest likely indicates that spillover from the forest occurs over large distances (at least 7km). Spillover effects were observed over 1km distances into oil palm in butterflies and ants in Borneo [16]. In our case, with increasing distance from forest, the forest community may be gradually replaced by species that sustain populations in non-forest landscapes or landscapes with highly fragmented forest. At least one species observed in oil palm *Eulaema nigrita*, is associated with non-forest landscapes [43]. Consequently, it is likely that movement patterns of species adapted to forest and the sorting of species adapted to oil palm habitats may be driving the pattern of decaying similarity with increasing distance from forest. The high mobility of orchid bees relative to other groups like ants makes quantifying the relative importance of these two mechanisms difficult [44]. However, the much lower overall bee abundances in oil palm suggests that even though they are permeable, oil palm plantations are not a high-quality matrix like some agroforestry systems such as shade-coffee [44,45].

Our results also generally support our second hypothesis that changes in community composition are associated with altered community phylogenetic and trait structure. The high phylogenetic evenness in the forest relative to oil palm suggests that environmental filtering may be involved in determining the structure of the oil palm community. Filtering is commonly observed in situations where communities assemble in relatively more stressful or novel environments [46]. The rarity of orchids and altered abiotic environment in oil palm may induce a strong environmental filter. In the case of tongue length, significant phylogenetic conservatism suggests that shorter tongue length in oil palm could be a consequence of phylogenetically-driven assembly. The tendency for species in oil palm to be more cosmopolitan in geographic range is consistent with a high proportion of tramp and invasive ant species observed in Southeast Asian oil palm [47]. Although this trait is not phylogenetically conserved, it may also be a consequence of assembly mechanisms involving filtering. 

 Our results are preliminary and should be interpreted with several caveats. As in other orchid bee studies [25,28], we cannot be certain that species-specific capture rates mirror natural abundances and our characterization of the environment is coarse relative to the precise physical and biochemical properties that may be critical to orchid bees. However, our sample size is comparable to other similar studies [28] and our results are intended to encourage more work on biodiversity in Neotropical oil palm. More investigation is needed into the management of local properties (e.g. epiphytes, [42]), however our study is part of a growing consensus that landscape-scale management of oil palm is critical for conservation [7] and the maintenance of ecosystem services [15]. Our results indicate that orchid bee community properties are increasingly impoverished at increasing distance from forest and that these changes likely have a phylogenetic and functional basis. 

## Supporting Information

Figure S1 **The proportion of individuals captured by time period.** Each period corresponds to approximately 80 minutes. Error bars show standard error.
(EPS)

Table S1 **Total captures for each species by region and attractant type.**
(PDF)

Table S2 **Total captures for each species by date, habitat, region and site.**
(PDF)

## References

[1] Sala OE (2000) Global Biodiversity Scenarios for the Year 2100&nbsp. Science 287: 1770–1774. doi:10.1126/science.287.5459.1770. PubMed: 10710299. 10710299

[2] Green RE, Cornell SJ, Scharlemann JPW, Balmford A (2005) Farming and the fate of wild nature. Science 307: 550–555. doi:10.1126/science.1106049. PubMed: 15618485. 15618485

[3] Gibbs HK, Ruesch AS, Achard F (2010) Tropical forests were the primary sources of new agricultural land in the 1980s and 1990s. Proc Natl Acad Sci U S A 107: 16732–16737. doi:10.1073/pnas.0910275107. PubMed: 20807750. 20807750 PMC2944736

[4] Rappole JH, King DI, Rivera J (2003) Coffee and conservation. Conserv Biol 17: 334–336. doi:10.1046/j.1523-1739.2003.01548.x.

[5] Phalan B, Bertzky M, Butchart SHM, Donald PF, Scharlemann JPW et al. (2013) Crop expansion and conservation priorities in tropical countries. PLOS ONE 8: e51759. doi:10.1371/journal.pone.0051759. PubMed: 23326316. 23326316 PMC3541398

[6] DeFries RS, Rudel T, Uriarte M, Hansen M (2010) Deforestation driven by urban population growth and agricultural trade in the twenty-first century. Nature Geosci 3: 178–181 doi:10.1038/ngeo756.

[7] Foster WA, Snaddon JL, Turner EC, Fayle TM, Cockerill TD et al. (2011) Establishing the Evidence Base for Maintaining Biodiversity and Ecosystem Function in the Oil Palm Landscapes of South East Asia. Philos Trans R Soc Lond B Biol Sci 366: 3277–3291. doi:10.1098/rstb.2011.0041. PubMed: 22006968. 22006968 PMC3179631

[8] Koh LP, Wilcove DS (2008) Is oil palm agriculture really destroying tropical biodiversity? Conserv Lett 1: 60–64. doi:10.1111/j.1755-263X.2008.00011.x.

[9] Turner EC, Snaddon JL, Fayle TM, Foster WA (2008) Oil Palm Research in Context: Identifying the Need for Biodiversity Assessment. PLOS ONE 3: e1572. doi:10.1371/journal.pone.0001572. PubMed: 18270566. 18270566 PMC2215746

[10] Bhagwat SA, Willis KJ, Birks HJ (2008) Agroforestry: a refuge for tropical biodiversity? Trends Ecol Evol 5: 261–267. PubMed: 18359125. 10.1016/j.tree.2008.01.005 18359125

[11] Kehlenbeck K, Maass BL (2004) Crop diversity and classification of homegardens in Central Sulawesi, Indonesia. Agroforest Syst 63: 53–62. doi:10.1023/B:AGFO.0000049433.95038.25.

[12] Danielsen F, Beukema H, Burgess ND, Parish F, Brühl CA et al. (2009) Biofuel plantations on forested lands: double jeopardy for biodiversity and climate. Conserv Biol 23: 348–358. doi:10.1111/j.1523-1739.2008.01096.x. PubMed: 19040648. 19040648

[13] Fitzherbert EB, Struebig MJ, Morel A, Danielsen F, Brühl CA et al. (2008) How will oil palm expansion affect biodiversity? Trends Ecol Evol 23: 538–545. doi:10.1016/j.tree.2008.06.012. PubMed: 18775582. 18775582

[14] Liow LH, Sodhi NS, Elmqvist T (2001) Bee diversity along a disturbance gradient in tropical lowland forests of south‐east Asia. J Appl Ecol 38: 180–192. doi:10.1046/j.1365-2664.2001.00582.x.

[15] Koh LP (2008) Birds defend oil palms from herbivorous insects. Ecol Appl 18: 821–825. doi:10.1890/07-1650.1. PubMed: 18536244. 18536244

[16] Lucey JM, Hill JK (2011) Spillover of Insects from Rain Forest into Adjacent Oil Palm Plantations. Biotropica 44: 368–377. doi:10.1111/j.1744-7429.2011.00824.x.

[17] Mayfield M (2005) The Importance of Nearby Forest to Known and Potential Pollinators of Oil Palm (Elaeis guineënsis Jacq.; Areceaceae) in southern Costa Rica. Econ Bot 59: 190–196. doi:10.1663/0013-0001(2005)059[0190:TIONFT]2.0.CO;2.

[18] Castiblanco C, Etter A, Aide TM (2013) Oil palm plantations in Colombia: a model of future expansion. Environl Sci Policy 27: 172–183. doi:10.1016/j.envsci.2013.01.003.

[19] Ramirez SR, Roubik DW, Skov C, Pierce NE (2010) Phylogeny, diversification patterns and historical biogeography of euglossine orchid bees (Hymenoptera: Apidae). Biol J Linn Soc Lond 100: 552–572. doi:10.1111/j.1095-8312.2010.01440.x.

[20] Roubik DW (1992) Ecology and natural history of tropical bees Cambridge. University of Cambridge Press. 504 pp. 10.1126/science.248.4958.1026 17745410

[21] Cameron SA (2004) Phylogeny and biology of neotropical orchid bees (Euglossini). Annu Rev Entomol 49: 377-404. doi:10.1146/annurev.ento.49.072103.115855. PubMed: 14651469. 14651469

[22] Webb CO, Ackerly DD, McPeek MA (2002) Phylogenies and community ecology. Annu Rev Ecol Evol Syst 33: 475-505. doi:10.1146/annurev.ecolsys.33.010802.150448.

[23] Mouquet N, Devictor V, Meynard CN, Munoz F, Bersier L-F et al. (2012) Ecophylogenetics: advances and perspectives. Biol Rev 87: 769–785. doi:10.1111/j.1469-185X.2012.00224.x. PubMed: 22432924. 22432924

[24] Thiele R (2005) Phenology and nest site preferences of wood-nesting bees in a Neotropical lowland rain forest. Stud Neotrop Fauna Environ 40: 39–48. doi:10.1080/01650520400025712.

[25] Zimmermann Y, Ramírez SR, Eltz T (2009) Chemical niche differentiation among sympatric species of orchid bees. Ecology 90: 2994–3008. doi:10.1890/08-1858.1. PubMed: 19967856. 19967856

[26] Dressler RL (1982) Biology of the orchid bees (Euglossini). Annu Rev Ecol Syst 13: 373-394. doi:10.1146/annurev.es.13.110182.002105.

[27] Janzen DH, DeVries PJ, Higgins ML, Kimsey LS (1982) Seasonal and site variation in Costa Rican euglossine bees at chemical baits in lowland deciduous and evergreen forests. Ecology 63: 66-74. doi:10.2307/1937032.

[28] Brosi BJ (2009) The effects of forest fragmentation on euglossine bee communities (Hymenoptera: Apidae: Euglossini). Biol Conserv 142: 414–423. doi:10.1016/j.biocon.2008.11.003.

[29] Kimsey LS (1980) The behaviour of male orchid bees (Apidae, Hymenoptera, Insecta) and the question of leks. Anim Behav 28: 996–1004. doi:10.1016/S0003-3472(80)80088-1.

[30] Landolt PJ (1998) Chemical attractant for trapping yellow- jackets Vespula germanica and Vespula pensylvanica (Hymenoptera: Vespidae). Environ Entomol 27: 1229-1234.

[31] Armbruster WS, Berg EE (1994) Thermal ecology of male euglossine bees in a tropical wet forest: fragrance foraging in relation to operative temperature. Biotropica 26: 50-60. doi:10.2307/2389110.

[32] Nemésio A (2012) Methodological concerns and challenges in ecological studies with orchid bees (Hymenoptera: Apidae: Euglossina) = Desafios metodológicos em estudos ecológicos com abelhas-das-orquídeas (Hymenoptera: apidae: euglossina). Biosci J 28.

[33] Kembel SW, Cowan PD, Helmus MR, Cornwell WK, Morlon H et al. (2010) Picante: R tools for integrating phylogenies and ecology. Bioinformatics 26: 1463–1464. doi:10.1093/bioinformatics/btq166. PubMed: 20395285. 20395285

[34] Blomberg SP, Garland T, Ives AR (2003) Testing for phylogenetic signal in comparative data: behavioral traits are more labile. Evolution 57: 717-745. doi:10.1111/j.0014-3820.2003.tb00285.x. PubMed: 12778543. 12778543

[35] Wang J-P (2010) Estimating species richness by a Poisson-compound gamma model. Biometrika 97: 727-740. doi:10.1093/biomet/asq026. PubMed: 22822253. 22822253 PMC3372246

[36] Cao Y, Williams WP, Bark AW (1997) Similarity measure bias in river benthic Aufwuchs community analysis. Water Environ Res 69: 95-106. doi:10.2175/106143097X125227.

[37] Oksanen J, Blanchet FG, Kindt R, Legendre P, Minchin PR et al. (2013) vegan: Community Ecology Package. R package version 2.0-6. Retrieved onpublished at whilst December year 1111 from http://CRAN.R-project.org/package=vegan.

[38] Wikelski M, Moxley J, Eaton-Mordas A, López-Uribe MM, Holland R et al. (2010) Large-Range Movements of Neotropical Orchid Bees Observed via Radio Telemetry. PLOS ONE 5: e10738. doi:10.1371/journal.pone.0010738. PubMed: 20520813. 20520813 PMC2877081

[39] Powell AH, Powell G (1987) Population dynamics of male euglossine bees in Amazonian forest fragments. Biotropica 19: 176-179. doi:10.2307/2388742.

[40] Ackerman JD, Mesler MR, Lu KL, Montalvo AM (1982) Food foraging behaviour of male euglossine bees: Vagabonds or trapliners? Biotropica 14: 241–248. doi:10.2307/2388080.

[41] Tonhasca A, Blackmer JL, Albuquerque GS (2002) Abundance and Diversity of Euglossine Bees in the Fragmented Landscape of the Brazilian Atlantic Forest1. Biotropica 34: 416–422. doi:10.1111/j.1744-7429.2002.tb00555.x.

[42] Koh LP (2008) Can oil palm plantations be made more hospitable for forest butterflies and birds? J Appl Ecol 45: 1002–1009. doi:10.1111/j.1365-2664.2008.01491.x.

[43] Nemésio A, Silveira FA (2006) Deriving ecological relationships from geographical correlations between host and parasitic species: an example with orchid bees. J Biogeogr 33: 91–97. doi:10.1111/j.1365-2699.2005.01370.x.

[44] Livingston G, Philpott SM; la Mora Rodriguez de A (2012) Do Species Sorting and Mass Effects Drive Assembly in Tropical Agroecological Landscape Mosaics? Biotropica doi:10.1111/j.1744-7429.2012.00894.x.

[45] Vandermeer J, Perfecto I (2007) The agricultural matrix and a future paradigm for conservation. Conserv Biol 21: 274–277. doi:10.1111/j.1523-1739.2006.00582.x. PubMed: 17298536. 17298536

[46] Godfree R, Lepschi B, Mallinson D (2009) Ecological filtering of exotic plants in an Australian sub-alpine environment. J Veg Sci 15: 227–236. doi:10.1111/j.1654-1103.2004.tb02257.x.

[47] Pfeiffer M, Cheng Tuck H, Lay Chong T (2008) Exploring arboreal ant community composition and co-occurrence patterns in plantations of oil palm Elaeis guineensisin Borneo and Peninsular Malaysia. Ecography 31: 21–32. doi:10.1111/j.2007.0906-7590.05172.x.

