# Supplementary material for: Conservation Value and Permeability of Neotropical Oil Palm Landscapes for Orchid Bees
Source: PLoS One. 2013 Oct 17;8(10):e78523. doi: 10.1371/journal.pone.0078523 (PMC3798381; doi:10.1371/journal.pone.0078523)
Supplement: Table S1 — Total captures for each species by region and attractant type. (PDF) [file pone.0078523.s002.pdf]

	Forest		Adjacent palm		Intermediate palm		Distant palm	
	**Cineole**	**MS^**	**Cineole**	**MS**	**Cineole**	**MS**	**Cineole**	**MS**
Euglossa bursigera	32	1	10	0				
Euglossa championi	22	1	7	0				
Euglossa cognata	0	2						
Euglossa cyanapsis			6	0	8	0	1	0
Euglossa cybelia	2	0						
Euglossa deceptrix	1	0	1	0	1	0		
Euglossa despecta	2	0	4	0				
Euglossa dodsoni	3	0	2	0	1	0		
Euglossa erythrochlora	0	6	0	3	2	1		
Euglossa flammea	9	0						
Euglossa gorgonensis	6	0	2	0				
Euglossa hansoni	22	0	3	0	1	0		
Euglossa heterosticta	1	1						
Euglossa imperialis	198	55	4	2	10	1	3	0
Euglossa mixta	1	0	0	4				
Euglossa purpurea	27	1	7	2	2	0		
Euglossa sapphirina	38	44	5	12	2	1		
Euglossa tridentata	31	0	27	3	35	0	15	0
Euglossa variabilis	2	0	0	1	6	0	2	0
Euglossa villosiventris	0	2						
Eulaema bombiformis	0	13			0	1		
Eulaema nigrita					1	0	1	0
Eulaema leucopyga	1	0						
Eulaema meriana	1	11	0	16	0	3	0	1
Exaerate smaragdina	2	3	22	9	15	4	2	0
Exaerete frontalis	7	0						

^Methyl salicitate
