# Supplementary material for: Conservation Value and Permeability of Neotropical Oil Palm Landscapes for Orchid Bees
Source: PLoS One. 2013 Oct 17;8(10):e78523. doi: 10.1371/journal.pone.0078523 (PMC3798381; doi:10.1371/journal.pone.0078523)
Supplement: Table S2 — Total captures for each species by date, habitat, region and site. (PDF) [file pone.0078523.s003.pdf]

Date (2012, m/d)	6/10	6/11	6/12	6/13	6/14	6/15	6/16	6/22	6/23	6/24	6/25	7/1	7/2	7/3	7/4	7/9	7/10	7/11	7/12
**Site (F=forest, P=palm)**	F	F	F	F	F	F	F	P	P	P	P	P	P	P	P	P	P	P	P
**Region**	PN Corcovado							Adjacent				Intermediate				Distant			
**Site**	1	2	3	4	5	6	7	1	2	3	4	1	2	3	4	1	2	3	4
Euglossa bursigera	15	17	14	1	1	2	3	2	1	2	5								
Euglossa championi	2	8	4		6	2	1	2	2	3									
Euglossa cognata	1	1																	
Euglossa cyanapsis									3	1	2	1	2	2	4			1	
Euglossa cybelia			1	1															
Euglossa deceptrix				1							1								
Euglossa despecta	2							3			1		1						
Euglossa dodsoni	1	3		1				1			1		1						
Euglossa erythrochlora	1	1	2	1			1	1			2	1			2				
Euglossa flammea	2	2			1		4												
Euglossa gorgonensis	1	2	1				2				2								
Euglossa hansoni	8	12			2		2				3				1				
Euglossa heterosticta	1	1																	
Euglossa imperialis	27	52	65	13	44	12	40	1	1		4	4	1	1	5		1	2	
Euglossa mixta						1		1	2		1								
Euglossa purpurea	11	15	10		1	2	3		3	3	3		1	1					
Euglossa sapphirina	15	10	16	2	21	12	6	5	2	1	9				3				
Euglossa tridentata	18	5	9	1	2	2		15	4	6	5	14	8	6	7		3	6	6
Euglossa variabilis	2							1				3	2	1			2		
Euglossa villosiventris	1		1																
Eulaema bombiformis	1	1	11											1					
Eulaema leucopyga						1													
Eulaema meriana	8	1	4	1	1	1	2	1	2	5	8	2		1				1	
Eulaema nigrita												1							1
Exaerate smaragdina	1				5				7	7	17		2	9	1	1	1		
Exaerete frontalis		2		3	1	1						7							
